# Modeling Adherence Interventions Among Youth with HIV in the United States: Clinical and Economic Projections

**DOI:** 10.1007/s10461-021-03169-0

**Published:** 2021-02-06

**Authors:** Anne M. Neilan, Audrey C. Bangs, Michael Hudgens, Kunjal Patel, Allison L. Agwu, Ingrid V. Bassett, Aditya H. Gaur, Emily P. Hyle, Catherine M. Crespi, Keith J. Horvath, Caitlin M. Dugdale, Kimberly A. Powers, H. Jonathon Rendina, Milton C. Weinstein, Rochelle P. Walensky, Kenneth A. Freedberg, Andrea L. Ciaranello

**Affiliations:** 1 Division of General Academic Pediatrics, Department of Pediatrics, Massachusetts General Hospital, 100 Cambridge Street, Suite 1600, Boston, MA 02114 USA; 2 Division of Infectious Diseases, Department of Medicine, Massachusetts General Hospital, Boston, MA USA; 3 Medical Practice Evaluation Center, Massachusetts General Hospital, Boston, MA USA; 4 Harvard Medical School, Boston, MA USA; 5 Department of Biostatistics, Gillings School of Global Public Health, University of North Carolina at Chapel Hill, Chapel Hill, NC USA; 6 Department of Epidemiology and Center for Biostatistics in AIDS Research, Harvard T.H. Chan School of Public Health, Boston, MA USA; 7 Department of Pediatrics and Medicine, Division of Infectious Diseases, Johns Hopkins University School of Medicine, Baltimore, MD USA; 8 Harvard University Center for AIDS Research, Cambridge, MA USA; 9 St. Jude’s Children’s Research Hospital, Memphis, TN USA; 10 Department of Biostatistics, Fielding School of Public Health, University of California Los Angeles, Los Angeles, CA USA; 11 Department of Psychology, San Diego State University, San Diego, CA USA; 12 Department of Epidemiology, Gillings School of Global Public Health, University of North Carolina at Chapel Hill, Chapel Hill, NC USA; 13 Department of Psychology, Hunter College of the City University of New York, New York, NY USA; 14 Department of Health Policy and Management, Harvard T.H. Chan School of Public Health, Boston, MA USA; 15 Division of General Internal Medicine, Department of Medicine, Massachusetts General Hospital, Boston, MA USA

**Keywords:** Adolescents, Young adults, HIV, Adherence, Intervention, Cost-effectiveness, Modeling

## Abstract

The Adolescent Medicine Trials Network for HIV/AIDS Interventions is evaluating treatment adherence interventions (AI) to improve virologic suppression (VS) among youth with HIV (YWH). Using a microsimulation model, we compared two strategies: standard-of-care (SOC) and a hypothetical 12-month AI that increased cohort-level VS in YWH in care by an absolute ten percentage points and cost $100/month/person. Projected outcomes included primary HIV transmissions, deaths and life-expectancy, lifetime HIV-related costs, and incremental cost-effectiveness ratios (ICERs, $/quality-adjusted life-year [QALY]). Compared to SOC, AI would reduce HIV transmissions by 15% and deaths by 12% at 12 months. AI would improve discounted life expectancy/person by 8 months at an added lifetime cost/person of $5,300, resulting in an ICER of $7,900/QALY. AI would be cost-effective at $2,000/month/person or with efficacies as low as a 1 percentage point increase in VS. YWH-targeted adherence interventions with even modest efficacy could improve life expectancy, prevent onward HIV transmissions, and be cost-effective.

**Supplementary Information:**

The online version of this article (10.1007/s10461-021-03169-0) contains supplementary material, which is available to authorized users.

## Resumen

La Red de Ensayos Médicos sobre Adolescentes para Realizar Intervenciones sobre el VIH/SIDA está evaluando intervenciones de adherencia (IAs) al tratamiento para mejorar la supresión virológica (SV) entre los jóvenes con VIH (JCV). Usando un modelo de microsimulación, comparamos dos estrategias: cuidado convencional (CC) y una intervención de adherencia hipotética durando 12 meses que aumentaría la SV a nivel de cohorte entre JCV en tratamiento por 10 puntos de porcentuales y que costaría US$ 100/mes/persona. Resultados proyectados incluyeron transmisiones de VIH primarias, muertes y esperanza de vida, costos de por vida asociados con el VIH, y razones incrementales de costo-efectividad (RICEs, $/año de vida ajustado por la calidad [AVAC]). Comparado al CC, la IA reduciría transmisiones de VIH por 15% y muertes por 12% a los 12 meses. La IA mejoraría esperanza de vida descontada/persona por 8 meses a un costo de por vida adicional/persona de US$ 5.300, resultando en una RICE de US$ 7.900/AVAC. La IA sería costo-efectiva a un costo de US$ 2.000/mes/persona o si mejorara SV por al menos un punto porcentual. Intervenciones de adherencia dirigidas a jóvenes con una eficacia incluso modesta podrían mejorar esperanza de vida, prevenir transmisiones de VIH, y ser costo-efectivas.

## Introduction

Despite the availability of tolerable and effective antiretroviral therapy (ART), estimated virologic suppression among US youth with HIV (YWH), including those undiagnosed and not in care, remains low, with estimates ranging from 12 to 27% [1, 2]. With over 50,000 YWH in the US [3], poor HIV control among YWH is an important clinical and public health issue. Compared to adults with HIV, YWH are less likely to know their HIV serostatus, to initiate HIV care and ART, and to remain in care [4]. YWH who face challenges with adhering to medications are at risk of developing viral resistance [5]. YWH without sustained viremia are at increased risk of disease progression, opportunistic infections, and may transmit HIV to others [6].

Adolescents with chronic illness, who transition from childhood to adulthood within a fractured health care system, fare more poorly than their adult counterparts [7]. For YWH, the challenges of experiencing adolescence with a chronic illness are further compounded by HIV-related stigma and negotiating new relationships, including intimate relationships while living with a sexually transmissible infection [4]. Youth-tailored interventions to improve HIV medication adherence, such as 2-way text messaging systems [8] and daily cell phone calls [9] have been effective at improving virologic suppression (with observed increases in virologic suppression of 9–36 percentage points at 6–12 months).

The Adolescent Medicine Trials Network for HIV/AIDS Interventions (ATN) is currently evaluating several interventions to improve ART adherence among YWH [10]. For proven and emerging strategies to be implemented at scale, program planners and policy makers will also need to understand the likely clinical outcomes and costs of any intervention [11]. Our objective was to model the short- and long-term clinical and economic impact of a hypothetical adherence intervention for YWH, in order to identify the efficacy, duration, and cost at which such interventions would provide good value.

## Methods

### Analytic Overview

Using the Cost-Effectiveness of Preventing AIDS Complications (CEPAC)-Adolescent model of HIV disease and treatment [12, 13], we simulate a closed cohort of YWH aged 13–24 years prescribed ART. Cohort demographics and other key input parameters are based on completed ATN studies and available published data among YWH in the US (Table 1). We compare two strategies: standard-of-care (SOC) and a 12-month hypothetical adherence intervention (AI) based on an interactive smartphone-based reminder system applied to everyone in the cohort. We model an AI that leads to an absolute increase in cohort-level virologic suppression (efficacy) of 10 percentage points compared to SOC (for example, the proportion of cohort with virologic suppression would increase from 50 to 60%) at 12 months and costs $100/person/month. Once the intervention ends, patients return to their individual baseline adherence level, although the clinical benefits of having achieved virologic suppression during the intervention can persist beyond the 12-month period (e.g. due higher CD4 count at the end of the intervention). We project HIV care continuum outcomes at cross-sectional time points, including proportions of the cohort alive, in care (attending a visit within the past 6 months), and virologically suppressed (viral load < 200 copies/mL). We also project opportunistic infections and primary HIV transmissions averted during the intervention, as well as life expectancy, the number needed to treat to prevent one HIV-related death, and lifetime HIV-related costs. We report incremental cost-effectiveness ratios (ICERs: the difference in cost divided by the difference in life expectancy between strategies) from the healthcare payer perspective; we also include in the ICER calculation the health and economic benefits attributable to the prevention of primary HIV transmissions during the 12 months of the adherence intervention. Because preference-based health-state utilities are not available for YWH, who may attach different values to health states compared to adults, we use utility weight data from adult studies to report ICERs in $/quality-adjusted life-year saved (QALY). We report clinical outcomes and costs, both undiscounted and discounted (3%/year); we defined a strategy as “cost-effective” if its ICER fell below a willingness-to-pay threshold of $100,000/QALY [14].

**Table 1:** Input parameters for a model of a 12-month adherence intervention in youth with HIV in the United States

Parameter	Base case value	Source
*Cohort characteristics*		
Age, mean (SD)	19.5 (3.6)	[21]
Male/Female sex, %	79/21	[69]
CD4 at model start, cells/µL, mean (SD)	545 (228)	[21]
HIV RNA setpoint off ART		[22]
Mean log_10_ copies/mL (copies/mL)	5.22 (165,800)	
Distribution, % of cohort		
> 100,000 copies/mL	25.1	
30,001–100,000	42.0	
10,001–30,000	20.9	
3,001–10,000	5.6	
501–3,000	6.4	
0–500	0	
*Baseline ART adherence*^*a*^* and virologic suppression*		
Adherence to ART ≤ 25 years, % of cohort		Modeled cohort^b^
Adherence > 90%	20	
Adherence 81–90%	14	
Adherence 71–80%	9	
Adherence 61–70%	7	
Adherence ≤ 60%	50	
Adherence to ART > 25 years, % of cohort		Modeled cohort^b^
Adherence > 90%	34	
Adherence 81–90%	12	
Adherence 71–80%	6	
Adherence 61–70%	5	
Adherence ≤ 60%	43	
ART efficacy (VL < 50 copies/mL at 48 weeks)^c^, %		
> 95% adherence	96.4	[25–28]
< 57% adherence	0	[29]
Late virologic failure, range by adherence level, monthly probability, %	0.2–18	[30–32]
*Loss to follow-up*		
Loss to follow-up after 12 months, range by adherence level, monthly probability	0.7–2	[21, 33–35]
Returning to care, monthly probability	0.015	[36]
*Opportunistic infections off ART, range by CD4 count, monthly probability*^*d*^		[70]
Pneumocystis pneumonia	0.0004–0.0084	
Mycobacterium avium complex	0.0001–0.0047	
Toxoplasmosis	0.0001–0.0007	
Cytomegalovirus	0.0001–0.0082	
Fungal infection	0.0001–0.0032	
Other opportunistic infection	0.0006–0.0116	
*Chronic AIDS death off ART, range by OI history, monthly probability*^*e*^		[70]
CD4 > 500	0.00025	
CD4 351–500	0.00583	
CD4 201–350	0.00092–0.02696	
CD4 101–200	0.00250–0.03303	
CD4 51–100	0.00341–0.03254	
CD4 0–50	0.01472–0.06900	
*Non-HIV-related death, by age, monthly probability*		[71, 72]
13–14 years	0.00001–0.00002	
15–19	0.00002–0.00004	
20–24	0.00003–0.00006	
25–29	0.00004–0.00007	
30–39	0.00005–0.00012	
40–49	0.00011–0.00025	
50–59	0.00027–0.00068	
60–69	0.00071–0.00175	
70–79	0.00181–0.00416	
80–99	0.00433–0.01320	
*HIV transmissions*		
HIV transmissions, range by VL, per 100PY		[20, 37, 38]
> 100,000 copies/mL	16.5	
10,001–100,000	14.8	
3,001–10,000	7.6	
501–3,000	3.8	
21–500	0.3	
0–20	0	
*Costs (USD 2018)*		
Adherence intervention, monthly	100	Modeled intervention
Routine care, range by CD4 cell count, monthly^f^	260–1,150	[40–42]
Opportunistic infection	7,100–16,700	[40–42]
ART, monthly	2,670	[43]

*SD* standard deviation, *HVL* HIV viral load (HIV RNA), *ART* antiretroviral therapy, *VS* virologic suppression, *USD* United States dollars, *PY* person-years

^a^Adherence is measured as percent of pills taken

^b^See Supplemental Methods for details

^c^Efficacy between 57 and 95% adherence is exponentially interpolated (Supplemental Methods)

^d^A multiplier of 0.2 is applied for patients on ART [73, 74]

^e^A multiplier of 0.1 is applied for patients on ART [73, 74]

^f^Higher CD4 counts are associated with LOWER routine care costs

Additional details of inputs may be found in the Supplemental Methods

### Model Structure

The CEPAC-Adolescent model is a validated Monte Carlo state-transition model of HIV disease and treatment [12, 13, 15, 16]. YWH enter the model between the ages of 13 and 24 and are simulated individually throughout their lifetimes. YWH experience user-specified monthly probabilities of clinical events, including loss to follow-up (LTFU), return to care, opportunistic infections (OIs), and mortality. At the end of the simulation, the model tallies clinical events, duration spent in each health state, life expectancies, and cost per-person. A technical description of the model is available online at https://www.massgeneral.org/medicine/mpec/research/cpac-model.

#### Natural History and Treatment

At model entry, YWH are assigned a CD4 count and HIV RNA from user-specified distributions, and all modeled YWH are prescribed ART. Effective ART leads to virologic suppression and increases in CD4 cell count. In the absence of effective ART, CD4 cell counts decline and HIV RNA increases to a viral load set point. ART effectiveness is influenced by each individual’s level of adherence to ART (Supplemental Methods). While data distinguishing between causes of viremia in YHIV are limited, resistance to newer antiretrovirals is uncommon among YWH [17, 18]; this analysis focuses on youth who lack virologic suppression due to adherence challenges. YWH with lower ART adherence experience lower probabilities of virologic suppression and greater monthly probabilities of becoming viremic after initially achieving suppression [19]. Those who become viremic also have the opportunity to re-suppress HIV RNA on the same ART regimen. To isolate the impact of the AI, YWH are assumed to remain in care for the 12-month intervention period (the AI duration) in both SOC and the AI, and thus do not experience LTFU during this time. After the intervention ends, YWH experience monthly probabilities of being LTFU, also stratified by adherence level, and while lost, are assumed to stop ART. YWH who are lost to follow-up may return to care at monthly probabilities or if they seek care after developing an opportunistic infection. YWH who re-initiate ART after returning to care can again achieve virologic suppression. Patients’ adherence is also specified to change as they age.

#### HIV Transmission

Members of the simulated cohort can transmit HIV to others during any month in which they are viremic [20]. The risk of onward HIV transmission is modeled as a function of HIV RNA level in any month; HIV RNA levels and thus transmission rates vary by response to ART. We compared the number of monthly primary transmissions (one generation) in both strategies for the duration of the intervention (12 months) to determine transmissions averted by the intervention. We estimate the benefits of averting transmissions by simulating two cohorts separately, one with HIV infection and one without HIV infection; both begin at the time transmission is assumed to occur in the cohort with HIV infection. We then calculate the difference in cost and life-years between a person with HIV infection at the time of transmission and someone without HIV infection and apply these life expectancy gains and cost savings to each transmission averted by the AI. People acquiring HIV are assumed to have the same demographic characteristics as index cases. People without HIV at the time of the modeled transmission event remain at risk for HIV acquisition later in life. Survival benefits and cost offsets of transmissions averted are discounted at 3%/year.

#### Adherence Intervention

With implementation of an AI, adherence to ART improves for the duration of the intervention, increasing the probability of virologic suppression and decreasing the monthly probability of later virologic failure (additional details in Supplemental Methods). Duration of the intervention and monthly cost throughout the intervention period can be varied. Efficacy is specified in terms of the increase in the proportion of the cohort achieving virologic suppression by intervention end, compared to the SOC cohort that does not receive the intervention.

### Model Inputs

#### Cohort Characteristics

Based on ATN and other published data, we modeled a population of YWH who were engaged in care and prescribed ART; published data included populations of youth with HIV acquired perinatally or non-perinatally. Mean age at model start is 19.5 years (SD 3.6, range 13–24 years), and 79% were male (Table 1). Mean CD4 count at model start is 545 cells/μL (SD 228), and virologic suppression is 50% [21, 22]. The distribution of adherence to ART in the cohort from ages 13–24 is derived from youth-specific literature [23]; after age 25, improvements in adherence are based on adult literature [24] and range by baseline adherence level (Supplemental Methods, Supplemental Table I).

#### Natural History, Treatment, and HIV Transmission

All YWH are prescribed current ART regimens with treatment efficacy based on dolutegravir-based ART (treatment efficacy: 96.4% at 48 weeks with ≥ 95% ART adherence) [25–28]. A minimum of 57% ART adherence is required to experience any possibility of initial virologic suppression [29]. Once suppressed on ART, YWH experience a monthly probability of subsequent virologic failure (range by adherence level: 0.2–18.0%) [30–32]. After the end of the AI, while engaged in HIV care, YWH experience a monthly probability of becoming lost to follow-up (0.7–2.0%) [33–35]. While lost to follow-up, YWH experience a 1.5% monthly probability of returning to HIV care, or 50% probability of return if they develop any opportunistic infection [36]. Transmission rates are 0.0–16.5 transmissions/100 person-years, depending on HIV RNA level (Table 1) [20, 37, 38].

#### Adherence Intervention

In the base case, we assume the hypothetical AI would increase absolute virologic suppression among the modeled cohort by an absolute increment of 10 percentage points above the levels expected with SOC by the end of the 12-month intervention [8, 9]. After the 12-month intervention period, adherence returns to baseline levels in the base case until they age to an improved adherence level at age 25 years. The base case AI cost of $100/month reflects the cost of an interactive smartphone-based reminder system [8, 39].

#### Costs

Routine HIV care costs are assumed to range from $260–1,150/month, depending on CD4 cell count [40–42]. The monthly cost of ART is estimated at $2,670/month [43, 44]; the full cost of ART is incurred regardless of adherence level.

#### Sensitivity Analyses and Additional Analyses

We varied key model input parameters to understand their impact on AI cost-effectiveness, including AI efficacy (absolute increases in virologic suppression of 1–15 percentage points), AI duration (3–24 months with a 10-percentage point increase in VS by intervention end), and AI costs ($50–2,000/person/month) to reflect a range of adherence interventions (*e.g.,* text-messaging systems, in-person counseling, and cash transfers) [39, 45–47]. We then varied combinations of cost and efficacy values together, to identify scenarios in which the ICER for the AI compared to SOC crossed the $100,000/QALY threshold or became cost-saving. Finally, we varied assumptions about the baseline adherence patterns at baseline and how these change with age.

## Results

### Clinical Outcomes: 12-Month and Lifetime Horizons

Over the 12-month intervention period, AI leads to lower rates of OIs (3.6 vs 4.0/100 person-years (PY), a decrease of 11%), HIV transmissions (6.9 vs. 8.1/100PY, a decrease of 15%), and deaths (1.3 vs. 1.5/100PY, a decrease of 12%, excluding those from averted HIV transmissions) compared to SOC (Table 2). To prevent one HIV-related death over one year, 556 YWH would need to receive the AI. Over the lifetime of the cohort, excluding life expectancy gains through aversion of primary HIV transmissions, AI would increase mean undiscounted life expectancy by 12 months (276 vs. 264 months), due to lasting improvements in CD4 counts and averted mortality within the main cohort.

**Table 2:** Clinical and cost-effectiveness outcomes for a model of a 12-month adherence intervention in youth with HIV in the United States compared to standard-of-care

	12-month outcomes Undiscounted			Lifetime outcomes Undiscounted		Lifetime outcomes Discounted		
Strategy	OIs (rate/100PY)	Onward HIV transmissions (rate/100PY)	Death (rate/100PY)	Life expectancy (months)	Per-person cost (USD)	Life expectancy (months)	Per-person cost (USD)	ICER ($/QALY)
SOC	4.0	8.1	1.5	264	778,900	151	453,500	–
AI	3.6	6.9	1.3	276	802,900	159	458,800	7,900

*OI* opportunistic infection; *SOC* standard-of-care; *AI* adherence intervention; *PY* person-year; *ICER* incremental cost-effectiveness ratio; *QALY* quality-adjusted life-year; USD 2018, 2018 US dollars

Where noted, life expectancy and costs are discounted at 3%/year. Costs and ICERs are rounded to the nearest $100. In-text cited costs are rounded separately. The ICER quantifies the cost-effectiveness of one strategy compared to another regarding the degree to which the intervention provides benefit relative to its cost. The willingness-to-pay-threshold is a normative value which varies widely by setting and decision-maker; for interpretability, we have chosen ≤$100,000/QALY, however a range of values have been suggested in US settings [14]

### HIV Care Continuum Outcomes at 1, 5, and 10 Years

**Fig. 1:**
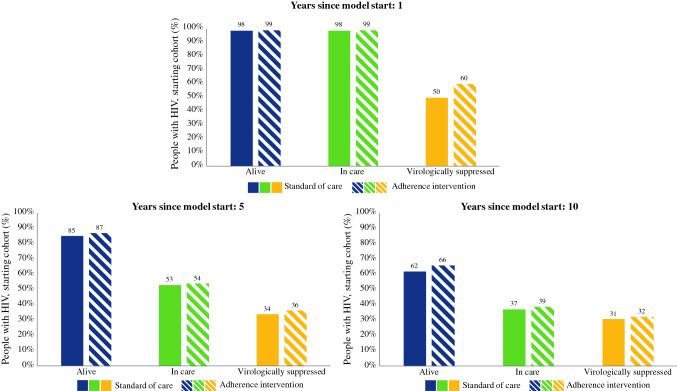
HIV care continuum outcomes: 12-month adherence intervention (AI) compared to standard-of-care (SOC). Includes cross-sectional snapshot of proportion alive, in care, and virologically suppressed of those in the initial cohort at A) one year after model start, B) five years after model start, and C) 10 years after model start. In both the cohorts, at model start, 100% of the modeled population was alive and in care, and 50% were virologically suppressed. AI began at model start and ended at Year 1. Years 5 and 10 therefore represent 4 and 9 years after completion of the intervention, respectively. Virologic suppression, among those in care, for SOC vs. AI was: Year 0: 50% vs. 50%; Year 1: 50% vs. 60%; Year 5: 64% vs. 67%; Year 10: 83% vs. 83%. *SOC* standard-of-care, *AI* adherence intervention

At 12 months after model start (the end of the intervention), an additional 1% of AI patients are alive and in care compared with SOC (99% vs. 98%), and an additional 10% of the entire cohort are virologically suppressed (Fig. 1, 60% vs. 50%). By 10 years after model start, an additional 4% and 2% of AI patients are alive (66% vs. 62%) and in care (39% vs. 37%) compared with SOC, respectively, and an additional 1% of the cohort (32% vs. 31%) is virologically suppressed on ART.

### Cost and Cost-Effectiveness

SOC would lead to lifetime discounted HIV-related costs of $453,500/person (Table 2). AI would increase discounted life expectancy by 8 months, at an additional discounted lifetime cost of $5300/person, resulting in an ICER of $7900/QALY. Excluding averted HIV transmissions, the ICER for AI would be $20,400/QALY (Supplemental Table 2). The difference in discounted cost between strategies ($5300/person) is the result of added costs (+ $11,900/person) being partially offset by cost-savings (− $6600/person). The added costs include the AI itself (+ $1,200/person, < 1% of the lifetime total cost of $458,800), as well as the cost of ART (+ $9,500/person) and HIV care (+ $1200/person) that result from longer survival. Of these added costs (+ $11,900/person), the intervention cost itself comprises 10%, while ART costs comprise 80%. The cost-savings (− $6600/person) result from averting HIV transmissions (− $5,700/person), opportunistic infections (− $500/person), and deaths (-$400/person).

### Sensitivity and Additional Analyses

**Fig. 2:**
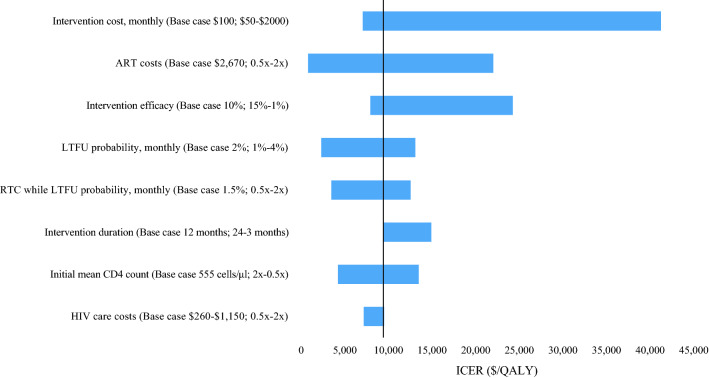
Sensitivity analyses: Incremental cost-effectiveness ratio of a 12-month adherence intervention (AI) compared to standard-of-care (SOC). Each parameter is varied through the range shown in parentheses, which is preceded by the base case input value. Incremental cost-effectiveness ratios (ICERs) for the comparison of adherence intervention to standard-of-care, in $/quality-adjusted life-year (QALY) are shown on the horizontal axis. The range of ICERs for each varied parameter is indicated by the blue horizontal bars. Longer blue horizontal bars indicate parameters to which the model results are more sensitive. The vertical black line represents the base case ICER. *ART* antiretroviral therapy, *LTFU* loss to follow-up, *ICER* incremental cost-effectiveness ratio, *RTC* return to care

In univariate sensitivity analyses, the ICER of AI compared to SOC is most sensitive to intervention cost, the efficacy of the intervention, and ART cost (Fig. 2). Varying the duration of the intervention compared to the base case would change the relative clinical and cost outcomes (Figure S1) but would have little impact on the ICER (Fig. 2). The value of AI would continue to improve if we further lengthened intervention duration (maintaining monthly costs and extending the duration of a 10% increase in virologic suppression by the end of the intervention) to 5 years (cost-saving), 10 years (cost-saving), and lifetime ($15,600/QALY).

**Fig. 3:**
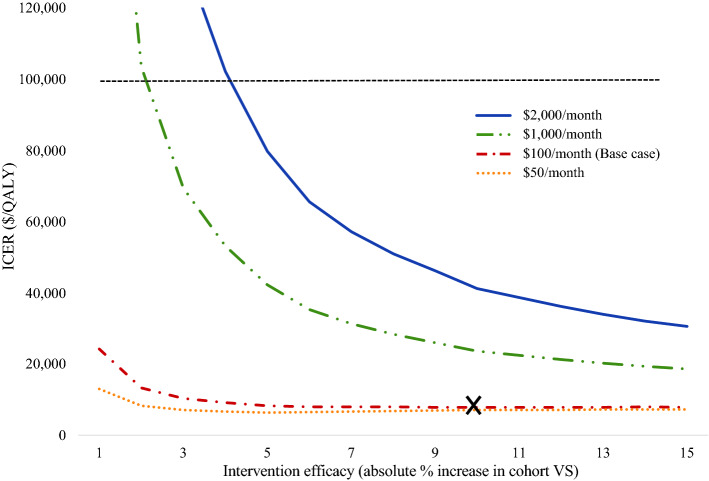
Two-way sensitivity analyses: varying adherence intervention cost and efficacy. Intervention efficacy and intervention cost were varied simultaneously. Intervention efficacy is displayed across the horizontal axis while intervention cost is shown as different series represented by color. Intervention efficacy is reported as an absolute increase in cohort-level virologic suppression in AI compared to SOC at the end of the intervention. The ICER produced is shown on the vertical axis in $/QALY. The base case is represented by an X, and the cost-effectiveness threshold is represented by a dashed horizontal line at $100,000/QALY. *ICER* incremental cost-effectiveness ratio, *QALY* quality-adjusted life-year, *VS* virologic suppression

If we vary intervention cost together with efficacy, the ICER would remain < $100,000/QALY at a wide combination of values (Fig. 3). At efficacies below 5%, however, the ICER would increase sharply with small increases in the monthly cost of the intervention. AI would become cost-saving if the cost of ART is reduced by at least 60% (Figures S2A and S2B). When we assume a baseline adherence pattern of a cohort with high virologic suppression (> 90%), the ICER would be $9,400/QALY. When we remove the assumption that adherence among YWH improves with age, the ICER would be $8,200/QALY.

## Discussion

The Adolescent Medicine Trials Network for HIV/AIDS Interventions is evaluating several technology-based interventions to improve ART adherence among youth with HIV. Using an adolescent-focused microsimulation model, our objective was to model the impact of hypothetical adherence interventions, based on the example of an interactive smartphone-based reminder system, to identify combinations of intervention characteristics that would render the intervention cost-effective for YWH across a lifetime.

We demonstrated that adherence interventions targeted to YWH to improve virologic suppression, if effective, could have a substantial impact on HIV transmissions, life expectancy, deaths, and costs. An adherence intervention that led to a 10 percentage point cohort-level increase in virologic suppression compared to the standard-of-care [8, 9] would decrease primary transmissions by 15% and deaths by 12% over the 12-month horizon of the intervention. The AI would increase projected overall life expectancy by 12 months, due to improvements in virologic suppression, and would lead to lasting clinical benefits (*i.e.,* fewer opportunistic infections and reduced mortality). These results build on findings from model-based studies of adherence interventions in adults, which have also reported increased adherence corresponding to virologic suppression [48], reduced transmissions [49, 50] and deaths [51], and increased life expectancy (range: 1.7–6.4 discounted quality-adjusted life-months) [16, 50, 52]. Our results suggest that investments in adherence interventions that improve virologic suppression, when implemented during adolescence and young adulthood, could have substantial impacts on long-term clinical outcomes.

While the AI would lead to a projected increase in cost of $5,300/person over a lifetime, only a small proportion of this increase was due to the cost of the intervention itself. The intervention-specific costs amounted to < 1% of a patient’s overall HIV-related lifetime costs. The greatest contributor to a patient’s lifetime cost was the cost of ART. Given the current high cost of ART in the US ($36,080-$48,000 annually in 2018) [53], any decrease in the cost of ART would improve the value of adherence interventions, since these interventions result in more people incurring the cost of ART who otherwise would not. When the cost of ART was reduced by half, the additional lifetime cost of the AI strategy decreased by 89% compared to SOC; these results suggest that efforts to reduce drug costs, such as improved access to generic ART, could further improve the value of adherence interventions.

We found that adherence interventions among youth could be cost-effective at a wide range of intervention effects on virologic suppression, particularly when the monthly per-person intervention cost was less than $500. Among other published cost-effectiveness analyses of adherence interventions for people with HIV (not necessarily specific to YWH), many report cost-effectiveness [16, 47, 48, 50, 52, 54, 55] or the potential for cost-effectiveness [51, 56, 57]. However, the existing body of literature on adherence interventions specifically for YWH remains limited [58–60]. Evaluations of adherence interventions in YWH, including cell phone calls or text messaging systems [8, 9, 39, 61–63], directly observed therapy [64] and social support systems [65], all report some level of feasibility and/or acceptability. Of these interventions, however, many remain untested in the setting of randomized, controlled clinical trials or implementation trials [39, 61–64]. Protocols currently underway in the ATN and elsewhere hold promise to provide valuable contributions to our current understanding of adherence interventions in YWH [10]. If these interventions are shown to be even modestly effective, our results suggest that they have the potential to improve individual- and population-level outcomes and could provide excellent value for money.

This analysis had several limitations. We made selected assumptions that may have led us to either over- or underestimate the clinical and economic value of the example adherence intervention. First, input values for adherence by age were derived separately from youth-specific and adult-specific literature. Although the trajectories of individuals’ adherence from childhood through adulthood are unknown, we assumed adherence improved in all people with HIV after age 25 on the basis of this literature [23, 24]. Second, we also assumed that the intervention had no lasting impact on adherence after the intervention ended. Removing either of these assumptions in sensitivity analyses did not change our conclusions. Third, detailed data are limited regarding the impact of adherence to virologic suppression for YWH with and without resistance. Data are also limited regarding the impact of adherence interventions among the poorest adherers (*e.g.* those who are ≤ 30% adherent at baseline) [29]. However, when we varied assumptions about the likelihood of YWH to virologically suppress, to derive any benefit from ART, and/or to become lost to follow-up, our conclusions remained unchanged. Finally, model parameters were derived from studies comprised of youth who acquired HIV both perinatally and non-perinatally; however, these groups have different clinical characteristics and associated resource utilization [17, 66]. While youth with perinatally-acquired HIV who age through adolescence into early adulthood face higher risks of viremia, advanced immunosuppression, HIV-associated illnesses, and mortality increases as they age [33, 67, 68], less is known regarding the long-term outcomes of youth with non-perinatally acquired HIV. Whenever feasible, data for youth with perinatally and non-perinatally acquired HIV should be reported separately, which would enable different projections for these distinct groups.

## Conclusions

We used a youth-focused microsimulation model of HIV disease and treatment to evaluate the clinical outcomes and cost-effectiveness of potential adherence interventions targeted towards youth with HIV. Intervention-associated increases in virologic suppression were projected to reduce opportunistic infections and mortality, improve life expectancy, and prevent transmission of HIV to sexual partners. We found that adherence interventions that prompt even small improvements in virologic suppression within a cohort of YWH could have a meaningful impact across a lifetime and be cost-effective.

## Supplementary Information

Below is the link to the electronic supplementary material.

Electronic supplementary material 1 (DOCX 356 kb)
