# Supplementary material for: Modeling Adherence Interventions Among Youth with HIV in the United States: Clinical and Economic Projections
Source: AIDS Behav. 2021 Feb 6;25(9):2973–84. doi: 10.1007/s10461-021-03169-0 (PMC8342630; doi:10.1007/s10461-021-03169-0)
Supplement: Supplementary file 1 — Electronic supplementary material 1 (DOCX 356 kb) [file 10461_2021_3169_MOESM1_ESM.docx]

**SUPPLEMENTAL APPENDIX**

**Modeling adherence interventions among youth with HIV in the United States:**

**clinical and economic projections**

Neilan, *et al.*

**TABLE OF CONTENTS**

**Supplemental Methods**

**Supplemental Table I.** Additional model input parameters for a model of a 12-month adherence intervention in youth with HIV in the United States

**Supplemental Table II.** Base case clinical and cost-effectiveness outcomes with and without the benefits of transmissions averted during the adherence intervention

**Figure S1.** One-way sensitivity analyses for an adherence intervention to improve virologic suppression among youth with HIV

**Figures S2a and S2b.** Two-way sensitivity analyses: a) Intervention costs and antiretroviral therapy costs b) Intervention efficacy and antiretroviral therapy costs

**Supplemental Methods**

*Base case cohort characteristics*

In the current Cost-effectiveness of Preventing AIDS Complications (CEPAC) model structure, all patients enter the model naïve to antiretroviral therapy (ART) and are initiated on ART in the first month of the model’s cycle. To generate a cohort of ART-experienced patients who then participate in an adherence intervention (AI), we first simulated an ART-naive “initialization” cohort with characteristics noted in manuscript Table 1 and Table S1 over a 1-year time horizon. At one year, we derived CD4 and adherence distributions separately for the two subgroups of the initialization cohort: those virologically suppressed and those not virologically suppressed at 1 year. The virologically suppressed sub-cohort had a history of recent virologic suppression and a higher mean CD4 cell count and adherence. The not virologically suppressed sub-cohort had a lower mean CD4 cell count and mean adherence. These two sub-cohorts thus had distinct CD4 cell counts, adherence distributions, and responses to antiretroviral therapy (*e.g*. CD4 cell count slopes). We weighted the results of the two sub-cohorts to create a single “time zero” cohort based on the percent of those who were vs. were not virologically suppressed at 1 year after initialization. The “time zero” cohort was then run with and without the intervention; all reported results are from combinations of the two cohorts weighted in this way.

*Adherence intervention*

Patients in the CEPAC models are assigned a “propensity to respond” (PTR) coefficient that simulates individual-level heterogeneity in patients’ responses to ART. PTR dictates adherence to ART and health-seeking behavior across each patient’s lifetime. It is drawn from a normal distribution defined by the user (Table I and Supplemental Table I) which is then logit-transformed to limit the range a value can assume from 0 to 1. This value represents percent adherence (proportion of days for which medication was prescribed over days between refills) which is used to relate adherence to probabilities of initial virologic suppression (one-time), virologic failure after initial suppression (monthly), and loss to follow-up (monthly). Users define the highest and lowest probabilities of each event (initial virologic suppression and subsequent failure and loss to follow-up), with exponential interpolation in between. This approach has been described elsewhere [3,4]. In this analysis, YWH in the AI strategy are assigned a PTR increase from their baseline adherence level that expires at the end of the intervention; this assumption is varied in sensitivity analysis. Individuals in the standard-of-care strategy did not receive a PTR coefficient increase during the intervention period. In the base case cohort, we also increase the PTR at age 25 in both the standard-of-care strategy and the adherence intervention, to reflect the assumption (varied in sensitivity analysis) that adherence improves with age; the 53 percentage point difference between the mean adherence level of the cohorts is maintained even when adherence improves at age 25.

| **Supplemental Table I.** Additional model input parameters for a model of a 12-month adherence intervention in youth with HIV in the United States | | | |
| --- | --- | --- | --- |
| Parameter | Base case value |  | Source |
| **Adherence to antiretroviral therapy, cohort distributions** | |  |  |
| Adherence to ART, % of initial cohort ^a^ | |  | [5,6] |
| Adherence >90% | 15 |  |  |
| Adherence 81–90% | 11 |  |  |
| Adherence 71–80% | 10 |  |  |
| Adherence 61–70% | 9 |  |  |
| Adherence ≤60% | 56 |  |  |
| **≤**25 years, % of virologically suppressed cohort |  |  | Modeled cohort |
| Adherence >90% | 40 |  |  |
| Adherence 81–90% | 27 |  |  |
| Adherence 71–80% | 15 |  |  |
| Adherence 61–70% | 8 |  |  |
| Adherence ≤60% | 10 |  |  |
| **≤**25 years, % of virologically non-suppressed cohort |  |  | Modeled cohort |
| Adherence >90% | 0 |  |  |
| Adherence 81–90% | 2 |  |  |
| Adherence 71–80% | 3 |  |  |
| Adherence 61–70% | 5 |  |  |
| Adherence ≤60% | 90 |  |  |

| **Supplemental Table I, continued.** Additional model input parameters for a model of adherence interventions in youth with HIV in the United States | | | |
| --- | --- | --- | --- |
| Parameter | Base case value |  | Source |
| >25 years, % of virologically suppressed cohort | |  | Modeled cohort |
| Adherence >90% | 67 |  |  |
| Adherence 81–90% | 20 |  |  |
| Adherence 71–80% | 7 |  |  |
| Adherence 61–70% | 3 |  |  |
| Adherence ≤60% | 2 |  |  |
| >25 years, % of virologically non-suppressed cohort | |  | Modeled cohort |
| Adherence >90% | 1 |  |  |
| Adherence 81–90% | 3 |  |  |
| Adherence 71–80% | 6 |  |  |
| Adherence 61–70% | 8 |  |  |
| Adherence ≤60% | 83 |  |  |
| **Quality-of-life, utility weights** | |  |  |
| Routine care, CD4 cell count |  |  | [7] |
| >500/μL | 0.87 |  |  |
| 201–500/μL | 0.86 |  |  |
| 51–200/μL | 0.85 |  |  |
| ≤50/μL | 0.83 |  |  |

| **Supplemental Table I, continued.** Additional model input parameters for a model of adherence interventions in youth with HIV in the United States | | | |
| --- | --- | --- | --- |
| Parameter | Base case value |  | Source |
| Acute opportunistic infections |  |  |  |
| Pneumocystis pneumonia | 0.74 |  | [8] |
| Mycobacterium Avium Complex | 0.69 |  |  |
| Toxoplasmosis | 0.69 |  |  |
| Cytomegalovirus | 0.78 |  |  |
| Severe fungal infection | 0.78 |  |  |
| Other opportunistic infection | 0.69 |  |  |
| ART: antiretroviral therapy; HVL: HIV viral load (HIV RNA)  ^a^ Applies to people initiating the model at ages 13-24. | | | |

| **Supplemental Table II.** Base case clinical and cost-effectiveness outcomes with and without the benefits of transmissions averted during the adherence intervention | | | | | | | |
| --- | --- | --- | --- | --- | --- | --- | --- |
|  | Without transmissions | | |  | With transmissions ^a^ | | |
| Strategy | Life expectancy  (months) | Per-person cost  (USD) | ICER  ($/QALY) |  | Life  expectancy  (months) | Per-person cost  (USD) | ICER  ($/QALY) |
| SOC | 151 | 453,500 | - |  | 151 | 453,500 | - |
| AI | 157 | 464,500 | 20,400 |  | 159 | 458,800 | 7,900 |
| SOC, standard-of-care; AI, adherence intervention; USD, 2018 US dollars; ICER, incremental cost-effectiveness ratio; QALY, quality-adjusted life-years.  Life expectancy and costs are discounted at 3%/year. Costs and ICERs are rounded to the nearest $100.  ^a^ Base case results shown in main manuscript. | | | | | | | |

**Supplemental Figure Legends**

**Figure S1**. One-way sensitivity analyses for an adherence intervention to improve virologic suppression among youth with HIV

Key sensitivity analyses affect the difference in clinical and cost outcomes between the adherence intervention (AI) and the standard-of-care (SOC), compared to the base case; we report the absolute percentage change in the base case difference. Each parameter is varied as shown in parentheses, followed by a semicolon and the base case input value. Three clinical outcomes (transmissions averted during the adherence intervention, deaths averted in the first 10 years of model simulation, and quality-adjusted years of life saved) and the additional cost of AI compared to SOC are shown. We report cost results calculated with undiscounted costs. More favorable outcomes of AI vs. SOC compared with the base case are indicated by increasingly bright shades of green (fewer transmissions and deaths, more quality-adjusted years of life saved, and lower costs); less favorable outcomes are indicated by increasingly dark shades of red (more transmissions and deaths, fewer years of life saved, and higher costs). No change in color indicates no change compared to the base case. ART efficacy with >95% adherence refers to the probability of initial virologic suppression at 48 weeks given >95% mean adherence to antiretroviral therapy. The clinical benefit of the AI compared to SOC would be >25% larger than the base case if AI efficacy improves, AI has a longer duration, or initial CD4 cell count is lower (top panel). The clinical benefits of AI compared to SOC would be >90% lower than in the base case if AI efficacy is lower than 10%. Halving ART costs would lead to the greatest reduction in AI additional costs (>25% reduction), while increasing monthly AI costs to $2,000/month would lead to the greatest increase (>90% increase) (bottom panel).

Abbreviations: AI, adherence intervention; ICER, incremental cost-effectiveness ratio; LTFU, loss to follow-up; ART, antiretroviral therapy; VS; virologic suppression

**Figures S2A and S2B.** Two-way sensitivity analyses: a) Intervention costs and antiretroviral therapy costs b) Intervention efficacy and antiretroviral therapy costs

A hypothetical 12-month adherence intervention (AI) designed to produce a 10% absolute increase in cohort-level virologic suppression among a cohort of adolescents and young adults ages 13-24 would result in improved life expectancy at a higher lifetime cost when compared to the standard-of-care (SOC). Dividing the difference in cost between strategies by the difference in life expectancy (both discounted at 3%/year) produces an incremental cost-effectiveness ratio (ICER) reported in $/quality-adjusted life-years (QALY). Heat maps visualize the results of two-way sensitivity analyses plotting the ICER of AI compared to SOC. AI would become cost-saving when the cost of antiretroviral therapy (ART) is reduced by 60-90% (x-axes) in conjunction with A) AI cost varying from 0-$300/monthly (y-axis), and B) AI efficacy varying from 3-15% (y-axis). ICERs shown range from ≤$50,000/QALY to cost-saving. The base case is represented by an “X”.

Abbreviations: AI, adherence intervention; ICER, incremental cost-effectiveness ratio; ART, antiretroviral therapy; VS; virologic suppression

**Figure S1.** One-way sensitivity analyses for an adherence intervention to improve virologic suppression among youth with HIV

**Figure S2**. Two-way sensitivity analyses: A) Intervention costs and antiretroviral therapy costs B) Intervention efficacy and antiretroviral therapy costs

A.

B.

**REFERENCES**

1. Van Dyke R, Patel K, Kagan R, Karalius B, Traite S, Meyer III W, et al. Antiretroviral drug resistance among children and youth in the United States with perinatal HIV. Clin Infect Dis. 2016;63:133–7.

2. Collins I, Foster C, Tostevin A, Tookey P, Riordan A, Dunn D, et al. Clinical status of adolescents with perinatal HIV at transfer to adult care in the UK/Ireland. Clin Infect Dis. 2017;64:1105–12.

3. Ross EL, Weinstein MC, Schackman BR, Sax PE, Paltiel AD, Walensky RP, et al. The clinical role and cost-effectiveness of long-acting antiretroviral therapy. Clin Infect Dis. 2015;60:1102–10.

4. Dugdale C, Phillips T, Myer L, Hyle E, Brittain K, Freedberg K, et al. Cost-effectiveness of integrating postpartum antiretroviral therapy and infant care into maternal & child health services in South Africa. PLoS ONE. PLoS One; 2019;14.

5. Sax PE, Meyers JL, Mugavero M, Davis KL. Adherence to antiretroviral treatment and correlation with risk of hospitalization among commercially insured HIV patients in the United States. PLoS One. 2012;7.

6. Becker SL, Dezii CM, Burtcel B, Kawabata H, Hodder S. Young HIV-infected adults are at greater risk for medication nonadherence. MedGenMed. 2002;4:21.

7. Schackman BR, Goldie SJ, Freedberg KA, Losina E, Brazier J, Weinstein MC. Comparison of health state utilities using community and patient preference weights derived from a survey of patients with HIV/AIDS. Med Decis Making. 2002;22:27–38.

8. Paltiel AD, Scharfstein JA, Seage GR, Losina E, Goldie SJ, Weinstein MC, et al. A Monte Carlo simulation of advanced HIV disease: application to prevention of CMV infection. Med Decis Making. 1998;18:S93-105.
